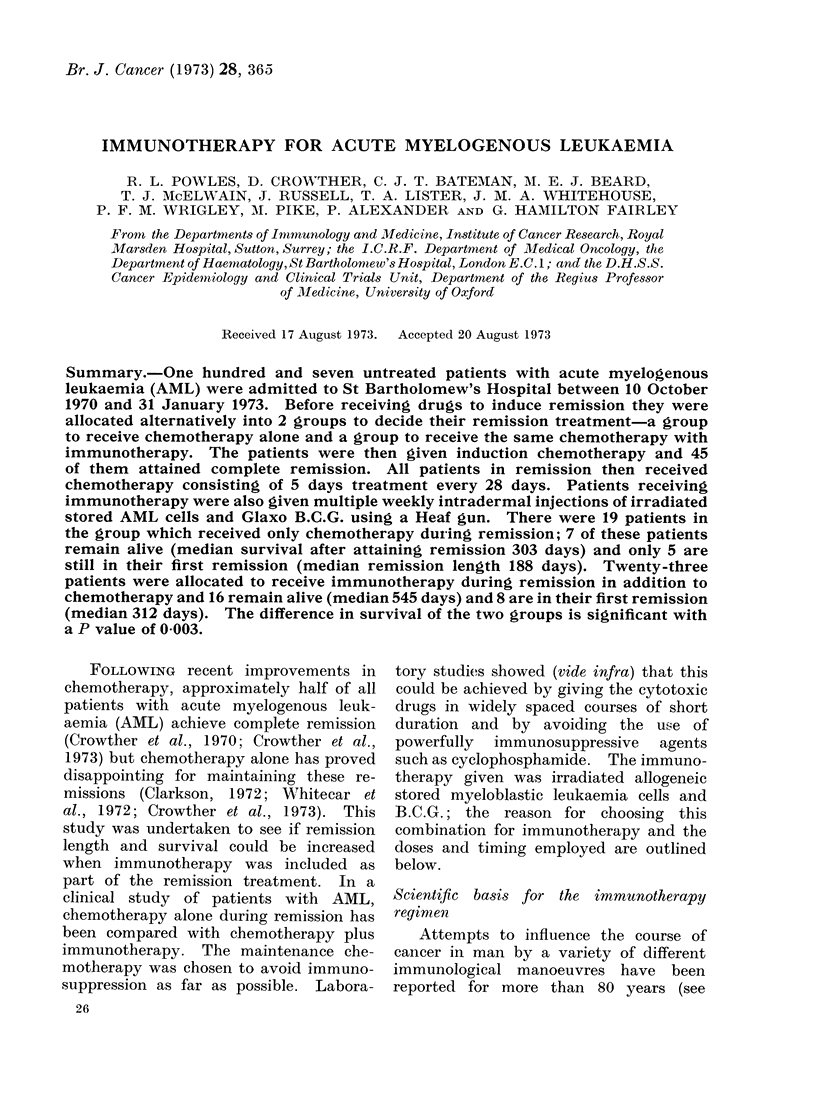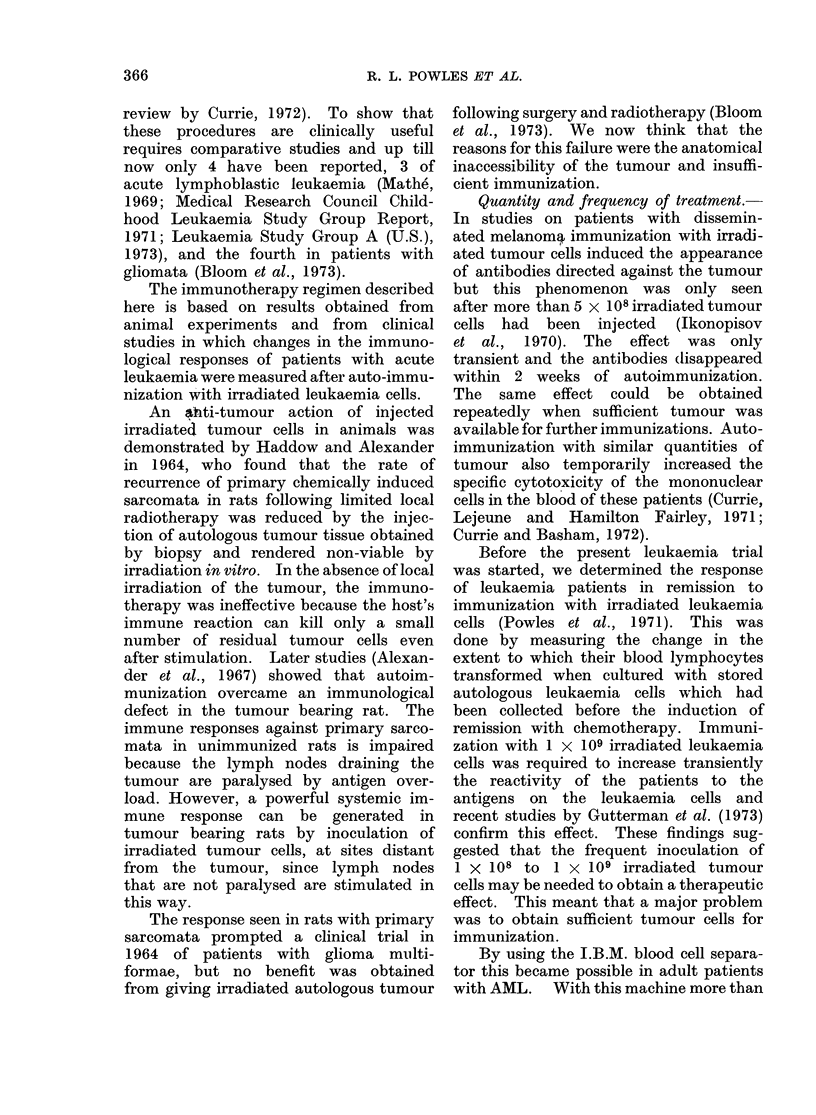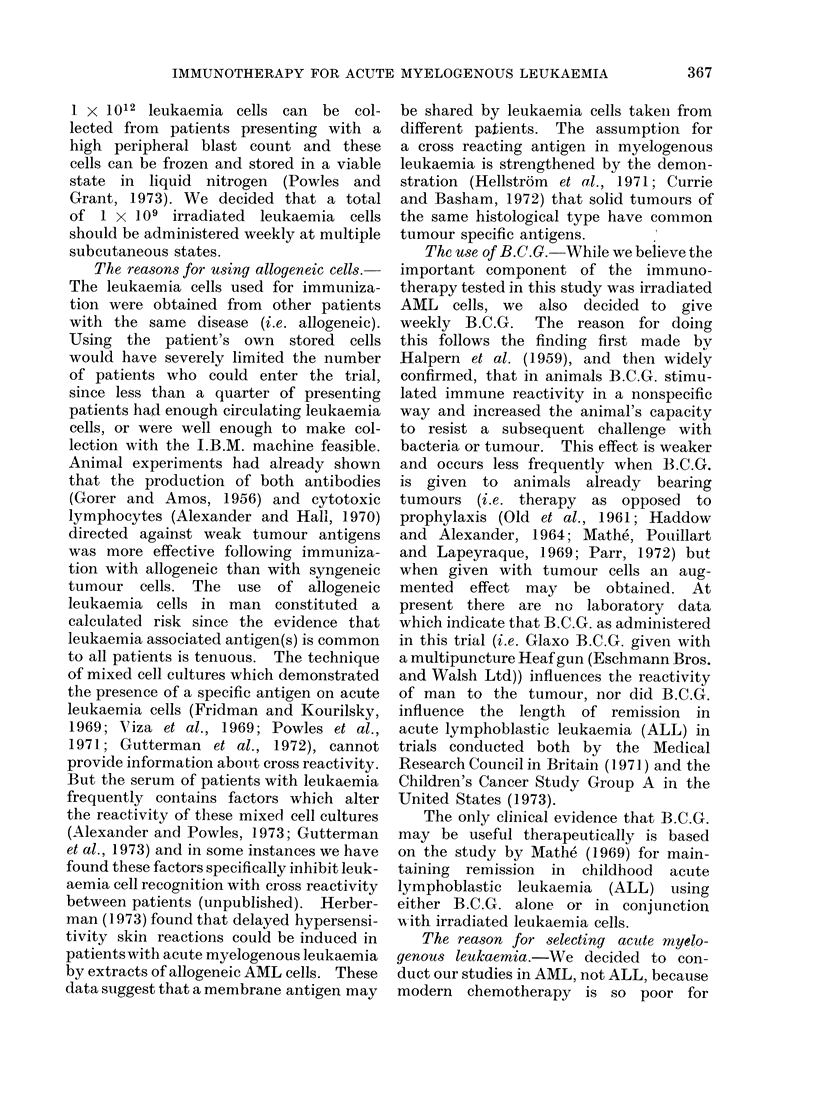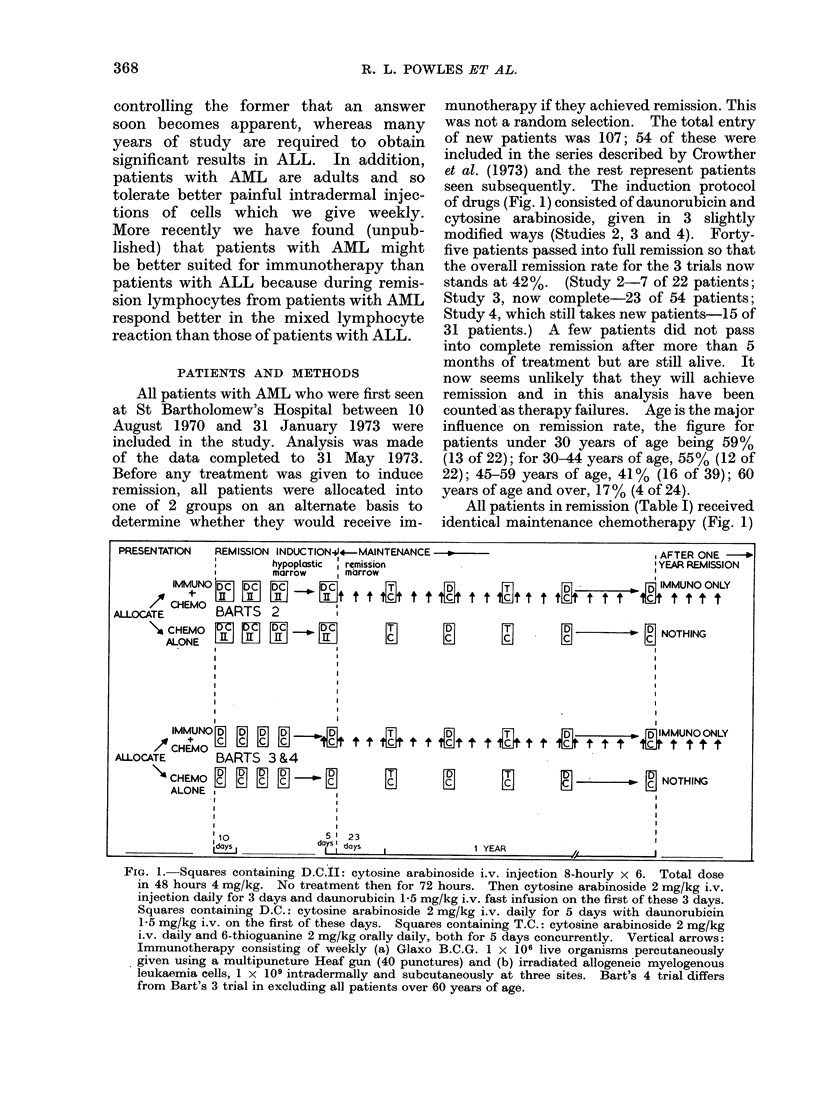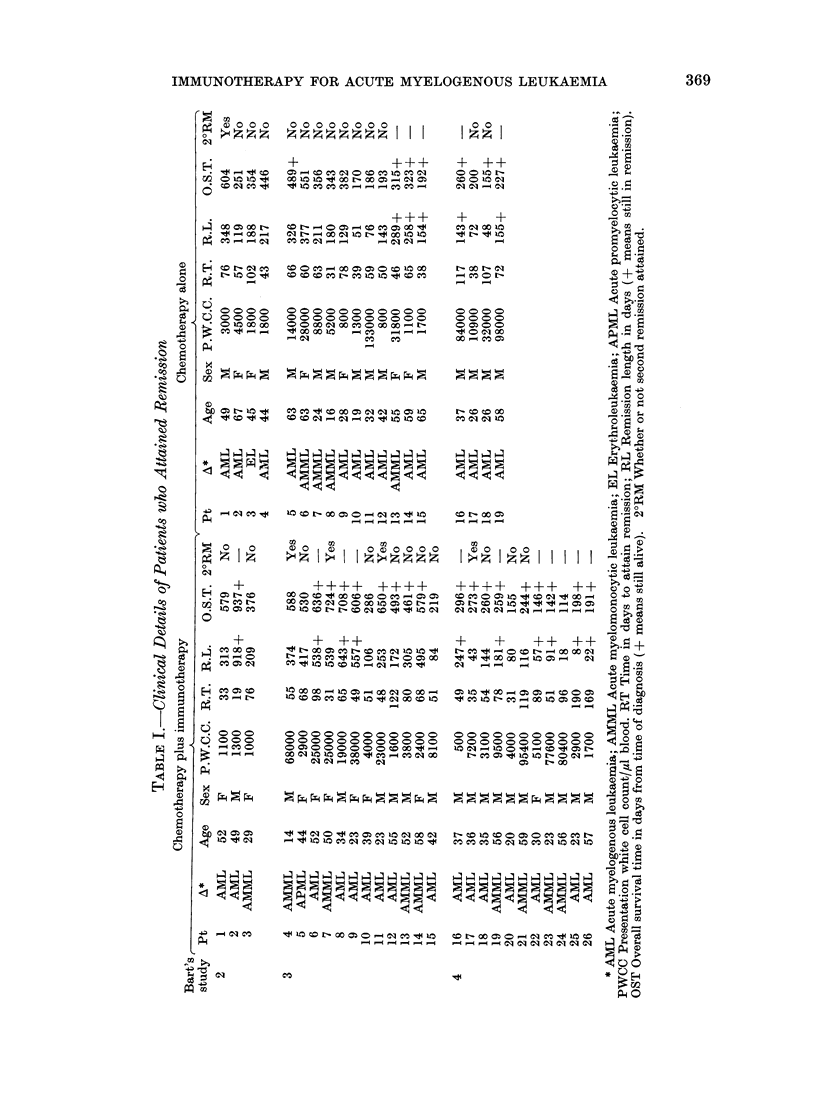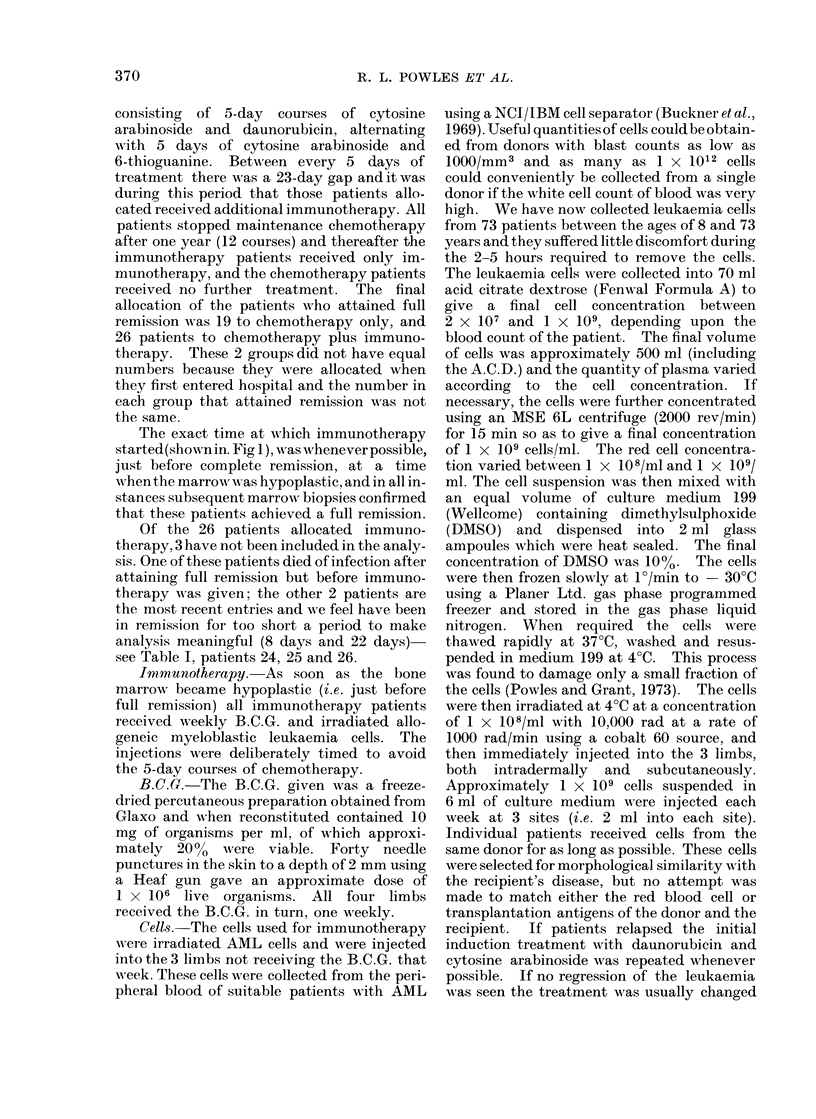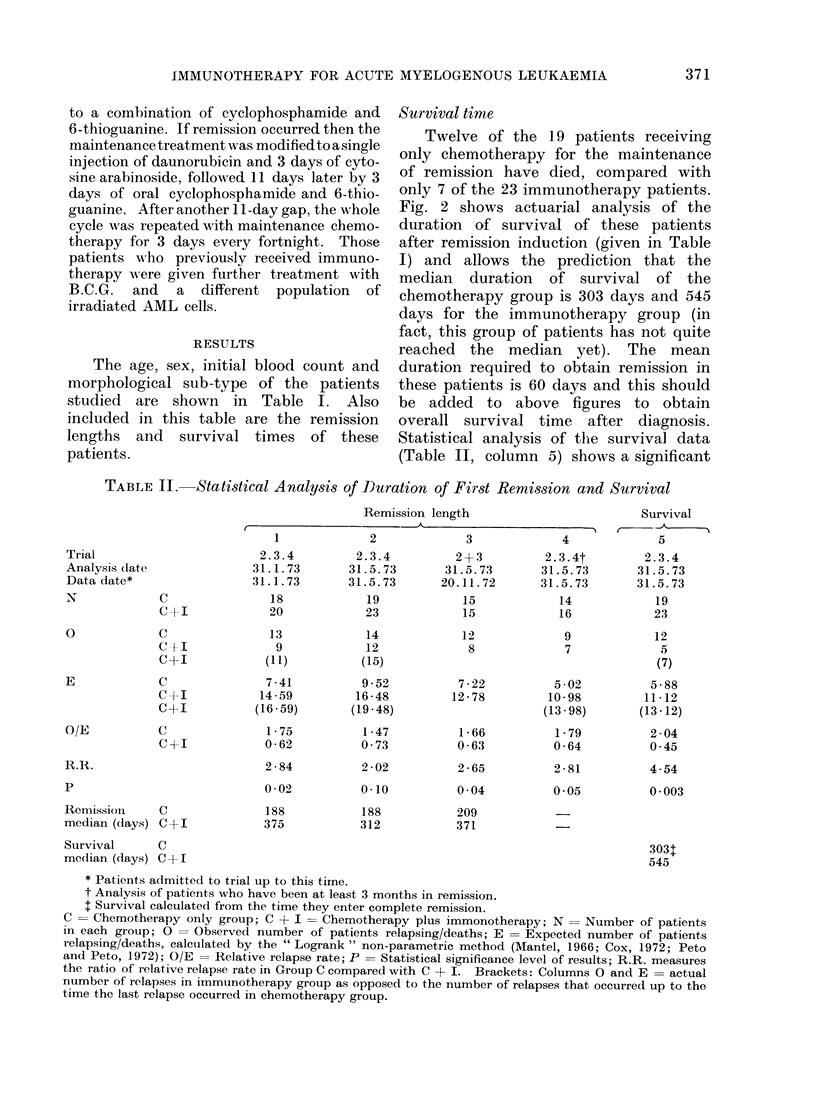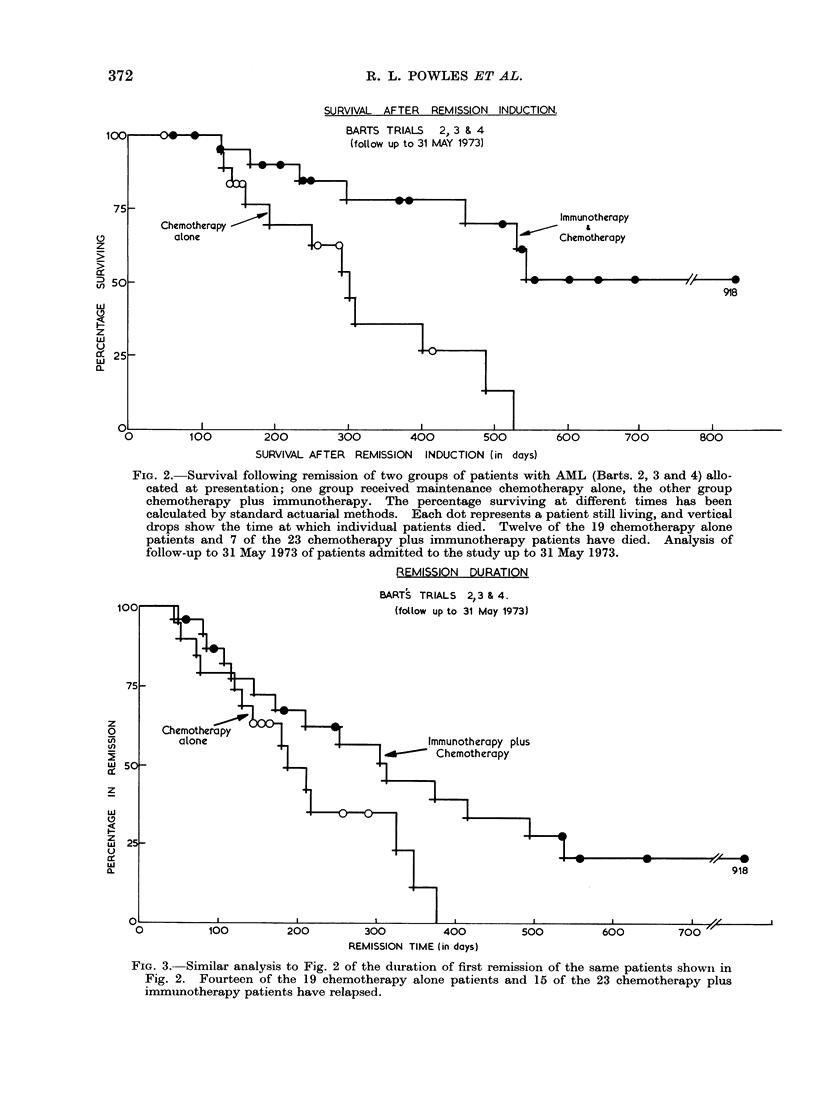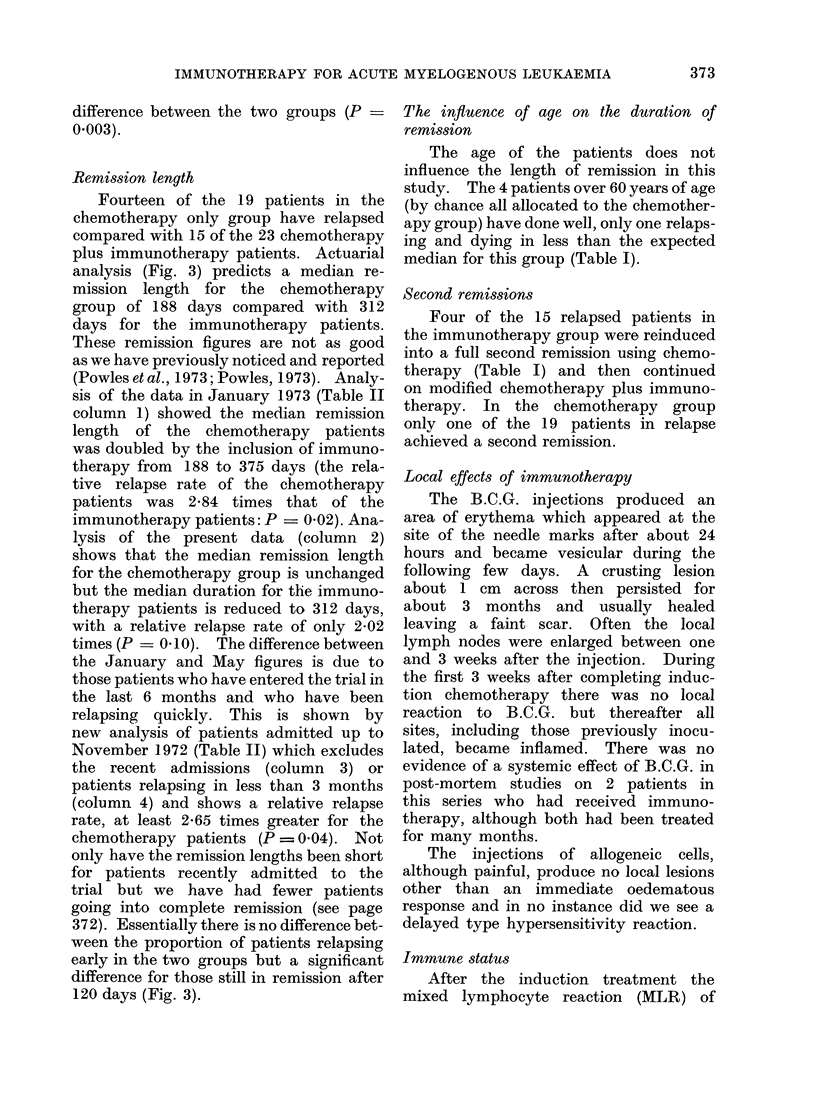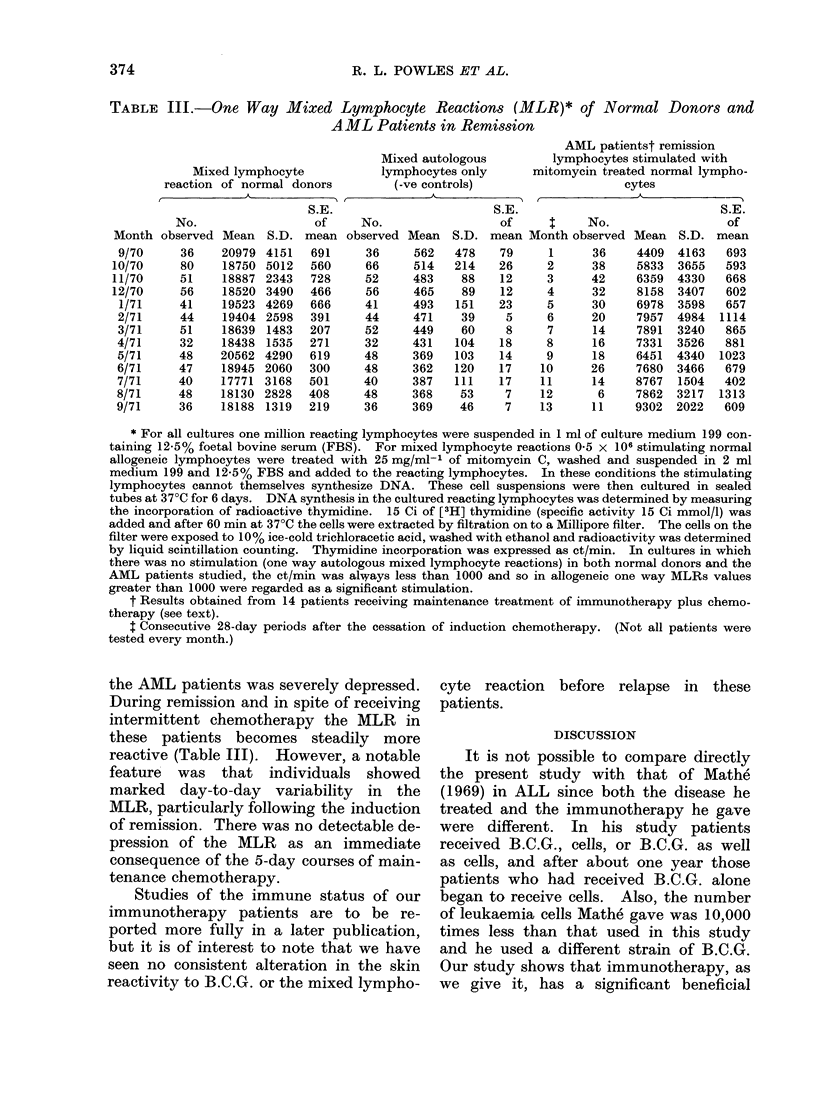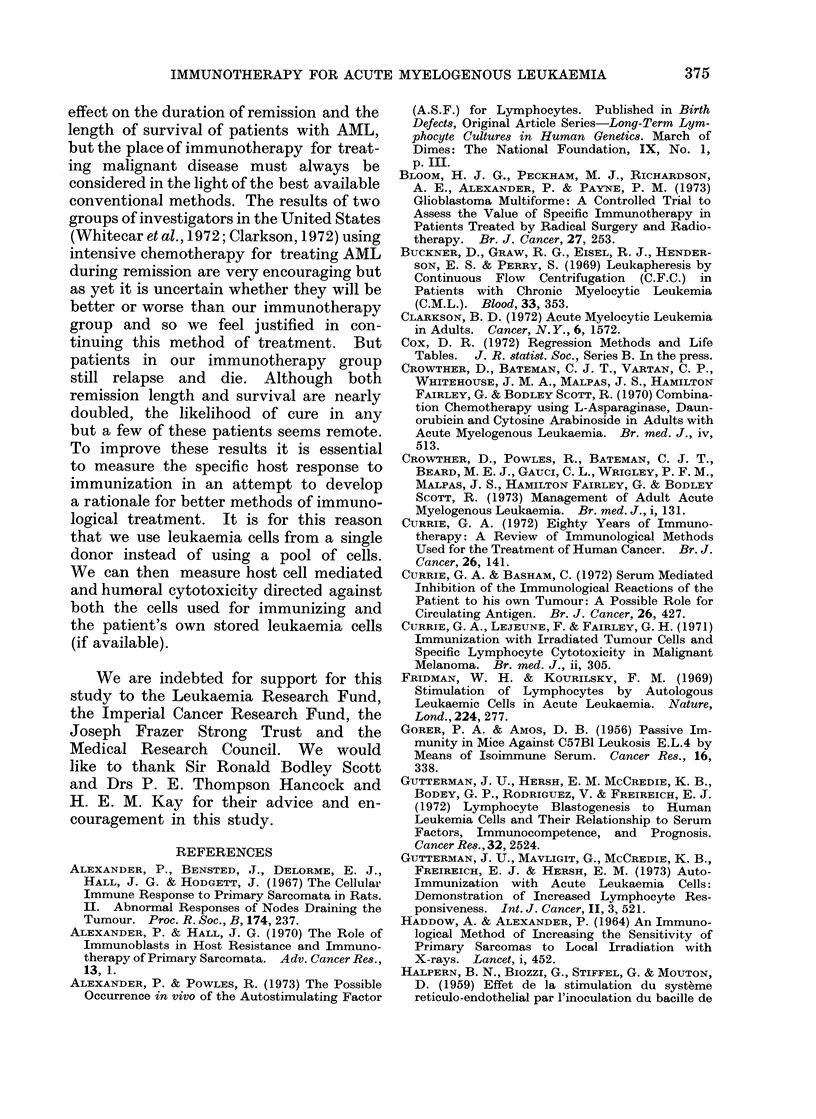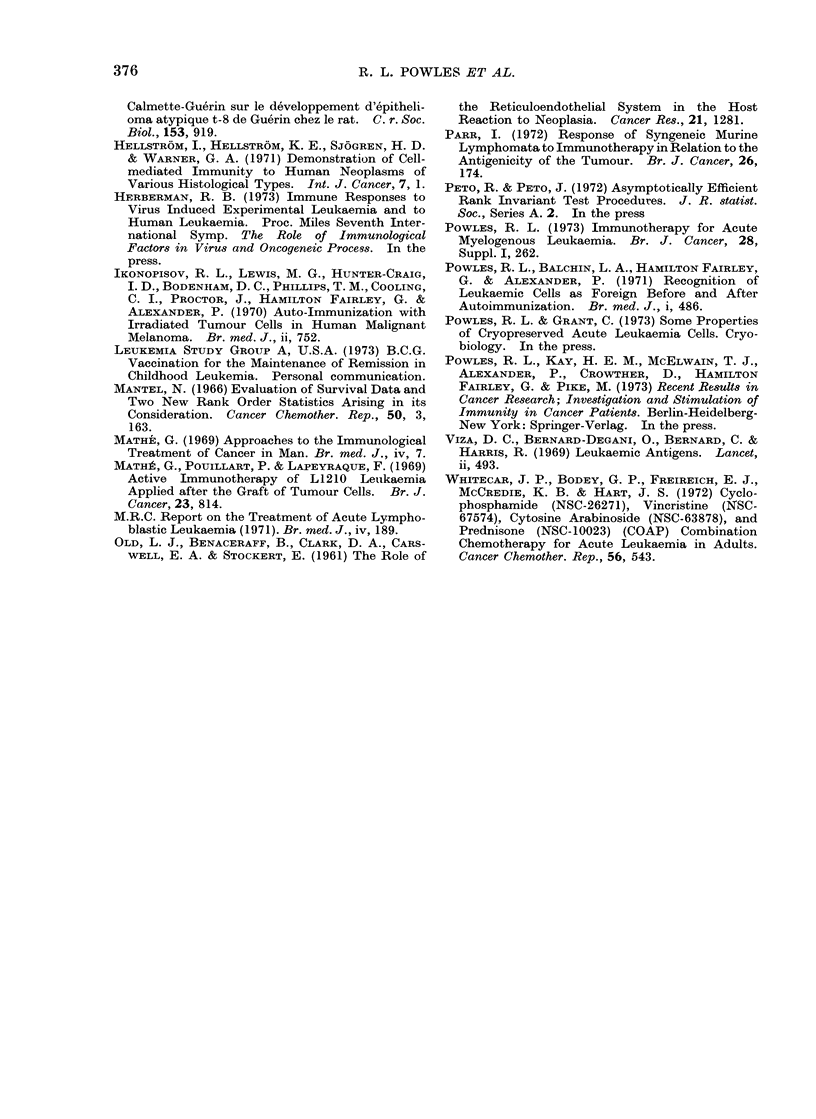# Immunotherapy for Acute Myelogenous Leukaemia

**DOI:** 10.1038/bjc.1973.162

**Published:** 1973-11

**Authors:** R. L. Powles, D. Crowther, C. J. T. Bateman, M. E. J. Beard, T. J. McElwain, J. Russell, T. A. Lister, J. M. A. Whitehouse, P. F. M. Wrigley, M. Pike, P. Alexander, G. Hamilton Fairley

## Abstract

One hundred and seven untreated patients with acute myelogenous leukaemia (AML) were admitted to St Bartholomew's Hospital between 10 October 1970 and 31 January 1973. Before receiving drugs to induce remission they were allocated alternatively into 2 groups to decide their remission treatment—a group to receive chemotherapy alone and a group to receive the same chemotherapy with immunotherapy. The patients were then given induction chemotherapy and 45 of them attained complete remission. All patients in remission then received chemotherapy consisting of 5 days treatment every 28 days. Patients receiving immunotherapy were also given multiple weekly intradermal injections of irradiated stored AML cells and Glaxo B.C.G. using a Heaf gun. There were 19 patients in the group which received only chemotherapy during remission; 7 of these patients remain alive (median survival after attaining remission 303 days) and only 5 are still in their first remission (median remission length 188 days). Twenty-three patients were allocated to receive immunotherapy during remission in addition to chemotherapy and 16 remain alive (median 545 days) and 8 are in their first remission (median 312 days). The difference in survival of the two groups is significant with a *P* value of 0·003.


*Br. J. Cancer* (1973) **28**, 365

### IMMUNOTHERAPY FOR ACUTE MYELOGENOUS LEUKAEMIA

R. L. POWLES, D. CROWTHER, C. J. T. BATEMAN, M. E. J. BEARD, T. J. McELWAIN, J. RUSSELL, T. A. LISTER, J. M. A. WHITEHOUSE, P. F. M. WRIGLEY, M. PIKE, P. ALEXANDER AND G. HAMILTON FAIRLEY

*From the Departments of Immunology and Medicine, Institute of Cancer Research, Royal Marsden Hospital, Sutton, Surrey; the I.C.R.F. Department of Medical Oncology, the Department of Haematology, St Bartholomew's Hospital, London E.C.1; and the D.H.S.S. Cancer Epidemiology and Clinical Trials Unit, Department of the Regius Professor of Medicine, University of Oxford*

Received 17 August 1973. Accepted 20 August 1973

**Summary.—One hundred and seven untreated patients with acute myelogenous leukaemia (AML) were admitted to St Bartholomew's Hospital between 10 October 1970 and 31 January 1973. Before receiving drugs to induce remission they were allocated alternatively into 2 groups to decide their remission treatment—a group to receive chemotherapy alone and a group to receive the same chemotherapy with immunotherapy. The patients were then given induction chemotherapy and 45 of them attained complete remission. All patients in remission then received chemotherapy consisting of 5 days treatment every 28 days. Patients receiving immunotherapy were also given multiple weekly intradermal injections of irradiated stored AML cells and Glaxo B.C.G. using a Heaf gun. There were 19 patients in the group which received only chemotherapy during remission; 7 of these patients remain alive (median survival after attaining remission 303 days) and only 5 are still in their first remission (median remission length 188 days). Twenty-three patients were allocated to receive immunotherapy during remission in addition to chemotherapy and 16 remain alive (median 545 days) and 8 are in their first remission (median 312 days). The difference in survival of the two groups is significant with a *P* value of 0·003.**

FOLLOWING recent improvements in chemotherapy, approximately half of all patients with acute myelogenous leukaemia (AML) achieve complete remission (Crowther *et al.*, 1970; Crowther *et al.*, 1973) but chemotherapy alone has proved disappointing for maintaining these remissions (Clarkson, 1972; Whitecar *et al.*, 1972; Crowther *et al.*, 1973). This study was undertaken to see if remission length and survival could be increased when immunotherapy was included as part of the remission treatment. In a clinical study of patients with AML, chemotherapy alone during remission has been compared with chemotherapy plus immunotherapy. The maintenance chemotherapy was chosen to avoid immunosuppression as far as possible. Laboratory studies showed (*vide infra*) that this could be achieved by giving the cytotoxic drugs in widely spaced courses of short duration and by avoiding the use of powerfully immunosuppressive agents such as cyclophosphamide. The immunotherapy given was irradiated allogeneic stored myeloblastic leukaemia cells and B.C.G.; the reason for choosing this combination for immunotherapy and the doses and timing employed are outlined below.

*Scientific basis for the immunotherapy regimen*

Attempts to influence the course of cancer in man by a variety of different immunological manoeuvres have been reported for more than 80 years (see review by Currie, 1972). To show that these procedures are clinically useful requires comparative studies and up till now only 4 have been reported, 3 of acute lymphoblastic leukaemia (Mathé, 1969; Medical Research Council Childhood Leukaemia Study Group Report, 1971; Leukaemia Study Group A (U.S.), 1973), and the fourth in patients with gliomata (Bloom *et al.*, 1973).

The immunotherapy regimen described here is based on results obtained from animal experiments and from clinical studies in which changes in the immunological responses of patients with acute leukaemia were measured after auto-immunization with irradiated leukaemia cells.

An anti-tumour action of injected irradiated tumour cells in animals was demonstrated by Haddow and Alexander in 1964, who found that the rate of recurrence of primary chemically induced sarcomata in rats following limited local radiotherapy was reduced by the injection of autologous tumour tissue obtained by biopsy and rendered non-viable by irradiation *in vitro*. In the absence of local irradiation of the tumour, the immunotherapy was ineffective because the host's immune reaction can kill only a small number of residual tumour cells even after stimulation. Later studies (Alexander *et al.*, 1967) showed that autoimmunization overcame an immunological defect in the tumour bearing rat. The immune responses against primary sarcomata in unimmunized rats is impaired because the lymph nodes draining the tumour are paralysed by antigen overload. However, a powerful systemic immune response can be generated in tumour bearing rats by inoculation of irradiated tumour cells, at sites distant from the tumour, since lymph nodes that are not paralysed are stimulated in this way.

The response seen in rats with primary sarcomata prompted a clinical trial in 1964 of patients with glioma multiformae, but no benefit was obtained from giving irradiated autologous tumour following surgery and radiotherapy (Bloom *et al.*, 1973). We now think that the reasons for this failure were the anatomical inaccessibility of the tumour and insufficient immunization.

*Quantity and frequency of treatment.*—In studies on patients with disseminated melanoma immunization with irradiated tumour cells induced the appearance of antibodies directed against the tumour but this phenomenon was only seen after more than $5 \times 10^8$ irradiated tumour cells had been injected (Ikonopisov *et al.*, 1970). The effect was only transient and the antibodies disappeared within 2 weeks of autoimmunization. The same effect could be obtained repeatedly when sufficient tumour was available for further immunizations. Autoimmunization with similar quantities of tumour also temporarily increased the specific cytotoxicity of the mononuclear cells in the blood of these patients (Currie, Lejeune and Hamilton Fairley, 1971; Currie and Basham, 1972).

Before the present leukaemia trial was started, we determined the response of leukaemia patients in remission to immunization with irradiated leukaemia cells (Powles *et al.*, 1971). This was done by measuring the change in the extent to which their blood lymphocytes transformed when cultured with stored autologous leukaemia cells which had been collected before the induction of remission with chemotherapy. Immunization with $1 \times 10^9$ irradiated leukaemia cells was required to increase transiently the reactivity of the patients to the antigens on the leukaemia cells and recent studies by Gutterman *et al.* (1973) confirm this effect. These findings suggested that the frequent inoculation of $1 \times 10^8$ to $1 \times 10^9$ irradiated tumour cells may be needed to obtain a therapeutic effect. This meant that a major problem was to obtain sufficient tumour cells for immunization.

By using the I.B.M. blood cell separator this became possible in adult patients with AML. With this machine more than

$1 \times 10^{12}$ leukaemia cells can be collected from patients presenting with a high peripheral blast count and these cells can be frozen and stored in a viable state in liquid nitrogen (Powles and Grant, 1973). We decided that a total of $1 \times 10^{9}$ irradiated leukaemia cells should be administered weekly at multiple subcutaneous states.

*The reasons for using allogeneic cells.*—The leukaemia cells used for immunization were obtained from other patients with the same disease (*i.e.* allogeneic). Using the patient's own stored cells would have severely limited the number of patients who could enter the trial, since less than a quarter of presenting patients had enough circulating leukaemia cells, or were well enough to make collection with the I.B.M. machine feasible. Animal experiments had already shown that the production of both antibodies (Gorer and Amos, 1956) and cytotoxic lymphocytes (Alexander and Hall, 1970) directed against weak tumour antigens was more effective following immunization with allogeneic than with syngeneic tumour cells. The use of allogeneic leukaemia cells in man constituted a calculated risk since the evidence that leukaemia associated antigen(s) is common to all patients is tenuous. The technique of mixed cell cultures which demonstrated the presence of a specific antigen on acute leukaemia cells (Fridman and Kourilsky, 1969; Viza *et al.*, 1969; Powles *et al.*, 1971; Gutterman *et al.*, 1972), cannot provide information about cross reactivity. But the serum of patients with leukaemia frequently contains factors which alter the reactivity of these mixed cell cultures (Alexander and Powles, 1973; Gutterman *et al.*, 1973) and in some instances we have found these factors specifically inhibit leukaemia cell recognition with cross reactivity between patients (unpublished). Herberman (1973) found that delayed hypersensitivity skin reactions could be induced in patients with acute myelogenous leukaemia by extracts of allogeneic AML cells. These data suggest that a membrane antigen may be shared by leukaemia cells taken from different patients. The assumption for a cross reacting antigen in myelogenous leukaemia is strengthened by the demonstration (Hellström *et al.*, 1971; Currie and Basham, 1972) that solid tumours of the same histological type have common tumour specific antigens.

*The use of B.C.G.*—While we believe the important component of the immunotherapy tested in this study was irradiated AML cells, we also decided to give weekly B.C.G. The reason for doing this follows the finding first made by Halpern *et al.* (1959), and then widely confirmed, that in animals B.C.G. stimulated immune reactivity in a nonspecific way and increased the animal's capacity to resist a subsequent challenge with bacteria or tumour. This effect is weaker and occurs less frequently when B.C.G. is given to animals already bearing tumours (*i.e.* therapy as opposed to prophylaxis (Old *et al.*, 1961; Haddow and Alexander, 1964; Mathé, Pouillart and Lapeyraque, 1969; Parr, 1972) but when given with tumour cells an augmented effect may be obtained. At present there are no laboratory data which indicate that B.C.G. as administered in this trial (*i.e.* Glaxo B.C.G. given with a multipuncture Heaf gun (Eschmann Bros. and Walsh Ltd)) influences the reactivity of man to the tumour, nor did B.C.G. influence the length of remission in acute lymphoblastic leukaemia (ALL) in trials conducted both by the Medical Research Council in Britain (1971) and the Children's Cancer Study Group A in the United States (1973).

The only clinical evidence that B.C.G. may be useful therapeutically is based on the study by Mathé (1969) for maintaining remission in childhood acute lymphoblastic leukaemia (ALL) using either B.C.G. alone or in conjunction with irradiated leukaemia cells.

*The reason for selecting acute myelogenous leukaemia.*—We decided to conduct our studies in AML, not ALL, because modern chemotherapy is so poor for controlling the former that an answer soon becomes apparent, whereas many years of study are required to obtain significant results in ALL. In addition, patients with AML are adults and so tolerate better painful intradermal injections of cells which we give weekly. More recently we have found (unpublished) that patients with AML might be better suited for immunotherapy than patients with ALL because during remission lymphocytes from patients with AML respond better in the mixed lymphocyte reaction than those of patients with ALL.

### PATIENTS AND METHODS

All patients with AML who were first seen at St Bartholomew's Hospital between 10 August 1970 and 31 January 1973 were included in the study. Analysis was made of the data completed to 31 May 1973. Before any treatment was given to induce remission, all patients were allocated into one of 2 groups on an alternate basis to determine whether they would receive immunotherapy if they achieved remission. This was not a random selection. The total entry of new patients was 107; 54 of these were included in the series described by Crowther *et al.* (1973) and the rest represent patients seen subsequently. The induction protocol of drugs (Fig. 1) consisted of daunorubicin and cytosine arabinoside, given in 3 slightly modified ways (Studies 2, 3 and 4). Forty-five patients passed into full remission so that the overall remission rate for the 3 trials now stands at 42%. (Study 2—7 of 22 patients; Study 3, now complete—23 of 54 patients; Study 4, which still takes new patients—15 of 31 patients.) A few patients did not pass into complete remission after more than 5 months of treatment but are still alive. It now seems unlikely that they will achieve remission and in this analysis have been counted as therapy failures. Age is the major influence on remission rate, the figure for patients under 30 years of age being 59% (13 of 22); for 30–44 years of age, 55% (12 of 22); 45–59 years of age, 41% (16 of 39); 60 years of age and over, 17% (4 of 24).

All patients in remission (Table I) received identical maintenance chemotherapy (Fig. 1)

FIG. 1.—Squares containing D.C.II: cytosine arabinoside i.v. injection 8-hourly × 6. Total dose in 48 hours 4 mg/kg. No treatment then for 72 hours. Then cytosine arabinoside 2 mg/kg i.v. injection daily for 3 days and daunorubicin 1·5 mg/kg i.v. fast infusion on the first of these 3 days. Squares containing D.C.: cytosine arabinoside 2 mg/kg i.v. daily for 5 days with daunorubicin 1·5 mg/kg i.v. on the first of these days. Squares containing T.C.: cytosine arabinoside 2 mg/kg i.v. daily and 6-thioguanine 2 mg/kg orally daily, both for 5 days concurrently. Vertical arrows: Immunotherapy consisting of weekly (a) Glaxo B.C.G. 1 × $10^6$ live organisms percutaneously given using a multipuncture Heaf gun (40 punctures) and (b) irradiated allogeneic myelogenous leukaemia cells, 1 × $10^9$ intradermally and subcutaneously at three sites. Bart's 4 trial differs from Bart's 3 trial in excluding all patients over 60 years of age.

TABLE I.—*Clinical Details of Patients who Attained Remission*

| Bart's | Chemotherapy plus immunotherapy | | | | | | | | | Chemotherapy alone | | | | | | | | | |
|---|---|---|---|---|---|---|---|---|---|---|---|---|---|---|---|---|---|---|---|
| study | Pt | Δ* | Age | Sex | P.W.C.C. | R.T. | R.L. | O.S.T. | 2°RM | Pt | Δ* | Age | Sex | P.W.C.C. | R.T. | R.L. | O.S.T. | 2°RM |
| 2 | 1 | AML | 52 | F | 1100 | 33 | 313 | 579 | No | 1 | AML | 49 | M | 3000 | 76 | 348 | 604 | Yes |
| | 2 | AML | 49 | M | 1300 | 19 | 918+ | 937+ | — | 2 | AML | 67 | F | 4500 | 57 | 119 | 251 | No |
| | 3 | AMML | 29 | F | 1000 | 76 | 209 | 376 | No | 3 | EL | 45 | F | 1800 | 102 | 188 | 354 | No |
| | | | | | | | | | | 4 | AML | 44 | M | 1800 | 43 | 217 | 446 | No |
| 3 | 4 | AMML | 14 | M | 68000 | 55 | 374 | 588 | Yes | 5 | AML | 63 | M | 14000 | 66 | 326 | 489+ | No |
| | 5 | APML | 44 | F | 2900 | 68 | 417 | 530 | No | 6 | AMML | 63 | F | 28000 | 60 | 377 | 551 | No |
| | 6 | AML | 52 | F | 25000 | 98 | 538+ | 636+ | — | 7 | AMML | 24 | M | 8800 | 63 | 211 | 356 | No |
| | 7 | AMML | 50 | F | 25000 | 31 | 539 | 724+ | Yes | 8 | AMML | 16 | M | 5200 | 31 | 180 | 343 | No |
| | 8 | AML | 34 | M | 19000 | 65 | 643+ | 708+ | — | 9 | AML | 28 | F | 800 | 78 | 129 | 382 | No |
| | 9 | AML | 23 | F | 38000 | 49 | 557+ | 606+ | — | 10 | AML | 19 | M | 1300 | 39 | 51 | 170 | No |
| | 10 | AML | 39 | F | 4000 | 51 | 106 | 286 | No | 11 | AML | 32 | M | 133000 | 59 | 76 | 186 | No |
| | 11 | AML | 23 | M | 23000 | 48 | 253 | 650+ | Yes | 12 | AML | 42 | M | 800 | 50 | 143 | 193 | No |
| | 12 | AML | 55 | M | 1600 | 122 | 172 | 493+ | No | 13 | AMML | 55 | F | 31800 | 46 | 289+ | 315+ | — |
| | 13 | AMML | 52 | M | 3800 | 80 | 305 | 461+ | No | 14 | AML | 59 | F | 1100 | 65 | 258+ | 323+ | — |
| | 14 | AMML | 58 | F | 2400 | 68 | 495 | 579+ | No | 15 | AML | 65 | M | 1700 | 38 | 154+ | 192+ | — |
| | 15 | AML | 42 | M | 8100 | 51 | 84 | 219 | No | | | | | | | | | |
| 4 | 16 | AML | 37 | M | 500 | 49 | 247+ | 296+ | — | 16 | AML | 37 | M | 84000 | 117 | 143+ | 260+ | — |
| | 17 | AML | 36 | M | 7200 | 35 | 43 | 273+ | Yes | 17 | AML | 26 | M | 10900 | 38 | 72 | 200 | No |
| | 18 | AML | 35 | M | 3100 | 54 | 144 | 260+ | No | 18 | AML | 26 | M | 32000 | 107 | 48 | 155+ | No |
| | 19 | AMML | 56 | M | 9500 | 78 | 181+ | 259+ | — | 19 | AML | 58 | M | 98000 | 72 | 155+ | 227+ | — |
| | 20 | AML | 20 | M | 4000 | 31 | 80 | 155 | No | | | | | | | | | |
| | 21 | AMML | 59 | M | 95400 | 119 | 116 | 244+ | No | | | | | | | | | |
| | 22 | AML | 30 | F | 5100 | 89 | 57+ | 146+ | — | | | | | | | | | |
| | 23 | AMML | 23 | M | 77600 | 51 | 91+ | 142+ | — | | | | | | | | | |
| | 24 | AMML | 56 | M | 80400 | 96 | 18 | 114 | — | | | | | | | | | |
| | 25 | AML | 23 | M | 2900 | 190 | 8+ | 198+ | — | | | | | | | | | |
| | 26 | AML | 57 | M | 1700 | 169 | 22+ | 191+ | — | | | | | | | | | |

* AML Acute myelogenous leukaemia; AMML Acute myelomonocytic leukaemia; EL Erythroleukaemia; APML Acute promyelocytic leukaemia; PWCC Presentation white cell count/μl blood. RT Time in days to attain remission; RL Remission length in days (+ means still in remission). OST Overall survival time in days from time of diagnosis (+ means still alive). 2°RM Whether or not second remission attained.

consisting of 5-day courses of cytosine arabinoside and daunorubicin, alternating with 5 days of cytosine arabinoside and 6-thioguanine. Between every 5 days of treatment there was a 23-day gap and it was during this period that those patients allocated received additional immunotherapy. All patients stopped maintenance chemotherapy after one year (12 courses) and thereafter the immunotherapy patients received only immunotherapy, and the chemotherapy patients received no further treatment. The final allocation of the patients who attained full remission was 19 to chemotherapy only, and 26 patients to chemotherapy plus immunotherapy. These 2 groups did not have equal numbers because they were allocated when they first entered hospital and the number in each group that attained remission was not the same.

The exact time at which immunotherapy started (shown in. Fig 1), was whenever possible, just before complete remission, at a time when the marrow was hypoplastic, and in all instances subsequent marrow biopsies confirmed that these patients achieved a full remission.

Of the 26 patients allocated immunotherapy, 3 have not been included in the analysis. One of these patients died of infection after attaining full remission but before immunotherapy was given; the other 2 patients are the most recent entries and we feel have been in remission for too short a period to make analysis meaningful (8 days and 22 days)—see Table I, patients 24, 25 and 26.

*Immunotherapy.*—As soon as the bone marrow became hypoplastic (*i.e.* just before full remission) all immunotherapy patients received weekly B.C.G. and irradiated allogeneic myeloblastic leukaemia cells. The injections were deliberately timed to avoid the 5-day courses of chemotherapy.

*B.C.G.*—The B.C.G. given was a freeze-dried percutaneous preparation obtained from Glaxo and when reconstituted contained 10 mg of organisms per ml, of which approximately 20% were viable. Forty needle punctures in the skin to a depth of 2 mm using a Heaf gun gave an approximate dose of $1 \times 10^6$ live organisms. All four limbs received the B.C.G. in turn, one weekly.

*Cells.*—The cells used for immunotherapy were irradiated AML cells and were injected into the 3 limbs not receiving the B.C.G. that week. These cells were collected from the peripheral blood of suitable patients with AML using a NCI/IBM cell separator (Buckner *et al.*, 1969). Useful quantities of cells could be obtained from donors with blast counts as low as $1000/mm^3$ and as many as $1 \times 10^{12}$ cells could conveniently be collected from a single donor if the white cell count of blood was very high. We have now collected leukaemia cells from 73 patients between the ages of 8 and 73 years and they suffered little discomfort during the 2–5 hours required to remove the cells. The leukaemia cells were collected into 70 ml acid citrate dextrose (Fenwal Formula A) to give a final cell concentration between $2 \times 10^7$ and $1 \times 10^9$, depending upon the blood count of the patient. The final volume of cells was approximately 500 ml (including the A.C.D.) and the quantity of plasma varied according to the cell concentration. If necessary, the cells were further concentrated using an MSE 6L centrifuge (2000 rev/min) for 15 min so as to give a final concentration of $1 \times 10^9$ cells/ml. The red cell concentration varied between $1 \times 10^8$/ml and $1 \times 10^9$/ml. The cell suspension was then mixed with an equal volume of culture medium 199 (Wellcome) containing dimethylsulphoxide (DMSO) and dispensed into 2 ml glass ampoules which were heat sealed. The final concentration of DMSO was 10%. The cells were then frozen slowly at 1°/min to − 30°C using a Planer Ltd. gas phase programmed freezer and stored in the gas phase liquid nitrogen. When required the cells were thawed rapidly at 37°C, washed and resuspended in medium 199 at 4°C. This process was found to damage only a small fraction of the cells (Powles and Grant, 1973). The cells were then irradiated at 4°C at a concentration of $1 \times 10^8$/ml with 10,000 rad at a rate of 1000 rad/min using a cobalt 60 source, and then immediately injected into the 3 limbs, both intradermally and subcutaneously. Approximately $1 \times 10^9$ cells suspended in 6 ml of culture medium were injected each week at 3 sites (*i.e.* 2 ml into each site). Individual patients received cells from the same donor for as long as possible. These cells were selected for morphological similarity with the recipient's disease, but no attempt was made to match either the red blood cell or transplantation antigens of the donor and the recipient. If patients relapsed the initial induction treatment with daunorubicin and cytosine arabinoside was repeated whenever possible. If no regression of the leukaemia was seen the treatment was usually changed to a combination of cyclophosphamide and 6-thioguanine. If remission occurred then the maintenance treatment was modified to a single injection of daunorubicin and 3 days of cytosine arabinoside, followed 11 days later by 3 days of oral cyclophosphamide and 6-thioguanine. After another 11-day gap, the whole cycle was repeated with maintenance chemotherapy for 3 days every fortnight. Those patients who previously received immunotherapy were given further treatment with B.C.G. and a different population of irradiated AML cells.

### RESULTS

The age, sex, initial blood count and morphological sub-type of the patients studied are shown in Table I. Also included in this table are the remission lengths and survival times of these patients.

*Survival time*

Twelve of the 19 patients receiving only chemotherapy for the maintenance of remission have died, compared with only 7 of the 23 immunotherapy patients. Fig. 2 shows actuarial analysis of the duration of survival of these patients after remission induction (given in Table I) and allows the prediction that the median duration of survival of the chemotherapy group is 303 days and 545 days for the immunotherapy group (in fact, this group of patients has not quite reached the median yet). The mean duration required to obtain remission in these patients is 60 days and this should be added to above figures to obtain overall survival time after diagnosis. Statistical analysis of the survival data (Table II, column 5) shows a significant

TABLE II.—*Statistical Analysis of Duration of First Remission and Survival*

| | | Remission length | | | | Survival |
|---|---|---|---|---|---|---|
| | | 1 | 2 | 3 | 4 | 5 |
| Trial | | 2.3.4 | 2.3.4 | 2+3 | 2.3.4† | 2.3.4 |
| Analysis date | | 31.1.73 | 31.5.73 | 31.5.73 | 31.5.73 | 31.5.73 |
| Data date* | | 31.1.73 | 31.5.73 | 20.11.72 | 31.5.73 | 31.5.73 |
| N | C | 18 | 19 | 15 | 14 | 19 |
| | C+I | 20 | 23 | 15 | 16 | 23 |
| O | C | 13 | 14 | 12 | 9 | 12 |
| | C+I | 9 | 12 | 8 | 7 | 5 |
| | C+I | (11) | (15) | | | (7) |
| E | C | 7·41 | 9·52 | 7·22 | 5·02 | 5·88 |
| | C+I | 14·59 | 16·48 | 12·78 | 10·98 | 11·12 |
| | C+I | (16·59) | (19·48) | | (13·98) | (13·12) |
| O/E | C | 1·75 | 1·47 | 1·66 | 1·79 | 2·04 |
| | C+I | 0·62 | 0·73 | 0·63 | 0·64 | 0·45 |
| R.R. | | 2·84 | 2·02 | 2·65 | 2·81 | 4·54 |
| P | | 0·02 | 0·10 | 0·04 | 0·05 | 0·003 |
| Remission | C | 188 | 188 | 209 | — | |
| median (days) | C+I | 375 | 312 | 371 | — | |
| Survival | C | | | | | 303‡ |
| median (days) | C+I | | | | | 545 |

* Patients admitted to trial up to this time.
† Analysis of patients who have been at least 3 months in remission.
‡ Survival calculated from the time they enter complete remission.
C = Chemotherapy only group; C + I = Chemotherapy plus immonotherapy; N = Number of patients in each group; O = Observed number of patients relapsing/deaths; E = Expected number of patients relapsing/deaths, calculated by the " Logrank " non-parametric method (Mantel, 1966; Cox, 1972; Peto and Peto, 1972); O/E = Relative relapse rate; *P* = Statistical significance level of results; R.R. measures the ratio of relative relapse rate in Group C compared with C + I. Brackets: Columns O and E = actual number of relapses in immunotherapy group as opposed to the number of relapses that occurred up to the time the last relapse occurred in chemotherapy group.

FIG. 2.—Survival following remission of two groups of patients with AML (Barts. 2, 3 and 4) allocated at presentation; one group received maintenance chemotherapy alone, the other group chemotherapy plus immunotherapy. The percentage surviving at different times has been calculated by standard actuarial methods. Each dot represents a patient still living, and vertical drops show the time at which individual patients died. Twelve of the 19 chemotherapy alone patients and 7 of the 23 chemotherapy plus immunotherapy patients have died. Analysis of follow-up to 31 May 1973 of patients admitted to the study up to 31 May 1973.

FIG. 3.—Similar analysis to Fig. 2 of the duration of first remission of the same patients shown in Fig. 2. Fourteen of the 19 chemotherapy alone patients and 15 of the 23 chemotherapy plus immunotherapy patients have relapsed.

difference between the two groups ($P$ = 0·003).

*Remission length*

Fourteen of the 19 patients in the chemotherapy only group have relapsed compared with 15 of the 23 chemotherapy plus immunotherapy patients. Actuarial analysis (Fig. 3) predicts a median remission length for the chemotherapy group of 188 days compared with 312 days for the immunotherapy patients. These remission figures are not as good as we have previously noticed and reported (Powles *et al.*, 1973; Powles, 1973). Analysis of the data in January 1973 (Table II column 1) showed the median remission length of the chemotherapy patients was doubled by the inclusion of immunotherapy from 188 to 375 days (the relative relapse rate of the chemotherapy patients was 2·84 times that of the immunotherapy patients: $P$ = 0·02). Analysis of the present data (column 2) shows that the median remission length for the chemotherapy group is unchanged but the median duration for the immunotherapy patients is reduced to 312 days, with a relative relapse rate of only 2·02 times ($P$ = 0·10). The difference between the January and May figures is due to those patients who have entered the trial in the last 6 months and who have been relapsing quickly. This is shown by new analysis of patients admitted up to November 1972 (Table II) which excludes the recent admissions (column 3) or patients relapsing in less than 3 months (column 4) and shows a relative relapse rate, at least 2·65 times greater for the chemotherapy patients ($P$ = 0·04). Not only have the remission lengths been short for patients recently admitted to the trial but we have had fewer patients going into complete remission (see page 372). Essentially there is no difference between the proportion of patients relapsing early in the two groups but a significant difference for those still in remission after 120 days (Fig. 3).

*The influence of age on the duration of remission*

The age of the patients does not influence the length of remission in this study. The 4 patients over 60 years of age (by chance all allocated to the chemotherapy group) have done well, only one relapsing and dying in less than the expected median for this group (Table I).

*Second remissions*

Four of the 15 relapsed patients in the immunotherapy group were reinduced into a full second remission using chemotherapy (Table I) and then continued on modified chemotherapy plus immunotherapy. In the chemotherapy group only one of the 19 patients in relapse achieved a second remission.

*Local effects of immunotherapy*

The B.C.G. injections produced an area of erythema which appeared at the site of the needle marks after about 24 hours and became vesicular during the following few days. A crusting lesion about 1 cm across then persisted for about 3 months and usually healed leaving a faint scar. Often the local lymph nodes were enlarged between one and 3 weeks after the injection. During the first 3 weeks after completing induction chemotherapy there was no local reaction to B.C.G. but thereafter all sites, including those previously inoculated, became inflamed. There was no evidence of a systemic effect of B.C.G. in post-mortem studies on 2 patients in this series who had received immunotherapy, although both had been treated for many months.

The injections of allogeneic cells, although painful, produce no local lesions other than an immediate oedematous response and in no instance did we see a delayed type hypersensitivity reaction.

*Immune status*

After the induction treatment the mixed lymphocyte reaction (MLR) of

TABLE III.—*One Way Mixed Lymphocyte Reactions (MLR)* of Normal Donors and AML Patients in Remission*

| | Mixed lymphocyte reaction of normal donors | | | | Mixed autologous lymphocytes only (-ve controls) | | | | AML patients† remission lymphocytes stimulated with mitomycin treated normal lymphocytes | | | | |
|---|---|---|---|---|---|---|---|---|---|---|---|---|---|
| Month | No. observed | Mean | S.D. | S.E. of mean | No. observed | Mean | S.D. | S.E. of mean | ‡ Month | No. observed | Mean | S.D. | S.E. of mean |
| 9/70 | 36 | 20979 | 4151 | 691 | 36 | 562 | 478 | 79 | 1 | 36 | 4409 | 4163 | 693 |
| 10/70 | 80 | 18750 | 5012 | 560 | 66 | 514 | 214 | 26 | 2 | 38 | 5833 | 3655 | 593 |
| 11/70 | 51 | 18887 | 2343 | 728 | 52 | 483 | 88 | 12 | 3 | 42 | 6359 | 4330 | 668 |
| 12/70 | 56 | 18520 | 3490 | 466 | 56 | 465 | 89 | 12 | 4 | 32 | 8158 | 3407 | 602 |
| 1/71 | 41 | 19523 | 4269 | 666 | 41 | 493 | 151 | 23 | 5 | 30 | 6978 | 3598 | 657 |
| 2/71 | 44 | 19404 | 2598 | 391 | 44 | 471 | 39 | 5 | 6 | 20 | 7957 | 4984 | 1114 |
| 3/71 | 51 | 18639 | 1483 | 207 | 52 | 449 | 60 | 8 | 7 | 14 | 7891 | 3240 | 865 |
| 4/71 | 32 | 18438 | 1535 | 271 | 32 | 431 | 104 | 18 | 8 | 16 | 7331 | 3526 | 881 |
| 5/71 | 48 | 20562 | 4290 | 619 | 48 | 369 | 103 | 14 | 9 | 18 | 6451 | 4340 | 1023 |
| 6/71 | 47 | 18945 | 2060 | 300 | 48 | 362 | 120 | 17 | 10 | 26 | 7680 | 3466 | 679 |
| 7/71 | 40 | 17771 | 3168 | 501 | 40 | 387 | 111 | 17 | 11 | 14 | 8767 | 1504 | 402 |
| 8/71 | 48 | 18130 | 2828 | 408 | 48 | 368 | 53 | 7 | 12 | 6 | 7862 | 3217 | 1313 |
| 9/71 | 36 | 18188 | 1319 | 219 | 36 | 369 | 46 | 7 | 13 | 11 | 9302 | 2022 | 609 |

* For all cultures one million reacting lymphocytes were suspended in 1 ml of culture medium 199 containing 12·5% foetal bovine serum (FBS). For mixed lymphocyte reactions $0{\cdot}5 \times 10^6$ stimulating normal allogeneic lymphocytes were treated with 25 $mg/ml^{-1}$ of mitomycin C, washed and suspended in 2 ml medium 199 and 12·5% FBS and added to the reacting lymphocytes. In these conditions the stimulating lymphocytes cannot themselves synthesize DNA. These cell suspensions were then cultured in sealed tubes at 37°C for 6 days. DNA synthesis in the cultured reacting lymphocytes was determined by measuring the incorporation of radioactive thymidine. 15 Ci of [$^3$H] thymidine (specific activity 15 Ci mmol/l) was added and after 60 min at 37°C the cells were extracted by filtration on to a Millipore filter. The cells on the filter were exposed to 10% ice-cold trichloracetic acid, washed with ethanol and radioactivity was determined by liquid scintillation counting. Thymidine incorporation was expressed as ct/min. In cultures in which there was no stimulation (one way autologous mixed lymphocyte reactions) in both normal donors and the AML patients studied, the ct/min was always less than 1000 and so in allogeneic one way MLRs values greater than 1000 were regarded as a significant stimulation.

† Results obtained from 14 patients receiving maintenance treatment of immunotherapy plus chemotherapy (see text).

‡ Consecutive 28-day periods after the cessation of induction chemotherapy. (Not all patients were tested every month.)

the AML patients was severely depressed. During remission and in spite of receiving intermittent chemotherapy the MLR in these patients becomes steadily more reactive (Table III). However, a notable feature was that individuals showed marked day-to-day variability in the MLR, particularly following the induction of remission. There was no detectable depression of the MLR as an immediate consequence of the 5-day courses of maintenance chemotherapy.

Studies of the immune status of our immunotherapy patients are to be reported more fully in a later publication, but it is of interest to note that we have seen no consistent alteration in the skin reactivity to B.C.G. or the mixed lymphocyte reaction before relapse in these patients.

### DISCUSSION

It is not possible to compare directly the present study with that of Mathé (1969) in ALL since both the disease he treated and the immunotherapy he gave were different. In his study patients received B.C.G., cells, or B.C.G. as well as cells, and after about one year those patients who had received B.C.G. alone began to receive cells. Also, the number of leukaemia cells Mathé gave was 10,000 times less than that used in this study and he used a different strain of B.C.G. Our study shows that immunotherapy, as we give it, has a significant beneficial effect on the duration of remission and the length of survival of patients with AML, but the place of immunotherapy for treating malignant disease must always be considered in the light of the best available conventional methods. The results of two groups of investigators in the United States (Whitecar *et al.*, 1972; Clarkson, 1972) using intensive chemotherapy for treating AML during remission are very encouraging but as yet it is uncertain whether they will be better or worse than our immunotherapy group and so we feel justified in continuing this method of treatment. But patients in our immunotherapy group still relapse and die. Although both remission length and survival are nearly doubled, the likelihood of cure in any but a few of these patients seems remote. To improve these results it is essential to measure the specific host response to immunization in an attempt to develop a rationale for better methods of immunological treatment. It is for this reason that we use leukaemia cells from a single donor instead of using a pool of cells. We can then measure host cell mediated and humoral cytotoxicity directed against both the cells used for immunizing and the patient's own stored leukaemia cells (if available).

We are indebted for support for this study to the Leukaemia Research Fund, the Imperial Cancer Research Fund, the Joseph Frazer Strong Trust and the Medical Research Council. We would like to thank Sir Ronald Bodley Scott and Drs P. E. Thompson Hancock and H. E. M. Kay for their advice and encouragement in this study.

### REFERENCES

ALEXANDER, P., BENSTED, J., DELORME, E. J., HALL, J. G. & HODGETT, J. (1967) The Cellular Immune Response to Primary Sarcomata in Rats. II. Abnormal Responses of Nodes Draining the Tumour. *Proc. R. Soc., B*, **174**, 237.

ALEXANDER, P. & HALL, J. G. (1970) The Role of Immunoblasts in Host Resistance and Immunotherapy of Primary Sarcomata. *Adv. Cancer Res.*, **13**, 1.

ALEXANDER, P. & POWLES, R. (1973) The Possible Occurrence *in vivo* of the Autostimulating Factor (A.S.F.) for Lymphocytes. Published in *Birth Defects*, Original Article Series—*Long-Term Lymphocyte Cultures in Human Genetics.* March of Dimes: The National Foundation, **IX**, No. 1, p. III.

BLOOM, H. J. G., PECKHAM, M. J., RICHARDSON, A. E., ALEXANDER, P. & PAYNE, P. M. (1973) Glioblastoma Multiforme: A Controlled Trial to Assess the Value of Specific Immunotherapy in Patients Treated by Radical Surgery and Radiotherapy. *Br. J. Cancer*, **27**, 253.

BUCKNER, D., GRAW, R. G., EISEL, R. J., HENDERSON, E. S. & PERRY, S. (1969) Leukapheresis by Continuous Flow Centrifugation (C.F.C.) in Patients with Chronic Myelocytic Leukemia (C.M.L.). *Blood*, **33**, 353.

CLARKSON, B. D. (1972) Acute Myelocytic Leukemia in Adults. *Cancer, N.Y.*, **6**, 1572.

COX, D. R. (1972) Regression Methods and Life Tables. *J. R. statist. Soc.*, Series B. In the press.

CROWTHER, D., BATEMAN, C. J. T., VARTAN, C. P., WHITEHOUSE, J. M. A., MALPAS, J. S., HAMILTON FAIRLEY, G. & BODLEY SCOTT, R. (1970) Combination Chemotherapy using L-Asparaginase, Daunorubicin and Cytosine Arabinoside in Adults with Acute Myelogenous Leukaemia. *Br. med. J.*, iv, 513.

CROWTHER, D., POWLES, R., BATEMAN, C. J. T., BEARD, M. E. J., GAUCI, C. L., WRIGLEY, P. F. M., MALPAS, J. S., HAMILTON FAIRLEY, G. & BODLEY SCOTT, R. (1973) Management of Adult Acute Myelogenous Leukaemia. *Br. med. J.*, i, 131.

CURRIE, G. A. (1972) Eighty Years of Immunotherapy: A Review of Immunological Methods Used for the Treatment of Human Cancer. *Br. J. Cancer*, **26**, 141.

CURRIE, G. A. & BASHAM, C. (1972) Serum Mediated Inhibition of the Immunological Reactions of the Patient to his own Tumour: A Possible Role for Circulating Antigen. *Br. J. Cancer*, **26**, 427.

CURRIE, G. A., LEJEUNE, F. & FAIRLEY, G. H. (1971) Immunization with Irradiated Tumour Cells and Specific Lymphocyte Cytotoxicity in Malignant Melanoma. *Br. med. J.*, ii, 305.

FRIDMAN, W. H. & KOURILSKY, F. M. (1969) Stimulation of Lymphocytes by Autologous Leukaemic Cells in Acute Leukaemia. *Nature, Lond.*, **224**, 277.

GORER, P. A. & AMOS, D. B. (1956) Passive Immunity in Mice Against C57Bl Leukosis E.L.4 by Means of Isoimmune Serum. *Cancer Res.*, **16**, 338.

GUTTERMAN, J. U., HERSH, E. M. MCCREDIE, K. B., BODEY, G. P., RODRIGUEZ, V. & FREIREICH, E. J. (1972) Lymphocyte Blastogenesis to Human Leukemia Cells and Their Relationship to Serum Factors, Immunocompetence, and Prognosis. *Cancer Res.*, **32**, 2524.

GUTTERMAN, J. U., MAVLIGIT, G., MCCREDIE, K. B., FREIREICH, E. J. & HERSH, E. M. (1973) Auto-Immunization with Acute Leukaemia Cells: Demonstration of Increased Lymphocyte Responsiveness. *Int. J. Cancer*, **II**, 3, 521.

HADDOW, A. & ALEXANDER, P. (1964) An Immunological Method of Increasing the Sensitivity of Primary Sarcomas to Local Irradiation with X-rays. *Lancet*, i, 452.

HALPERN, B. N., BIOZZI, G., STIFFEL, G. & MOUTON, D. (1959) Effet de la stimulation du systême reticulo-endothelial par l'inoculation du bacille de

Calmette-Guérin sur le développement d'épithelioma atypique t-8 de Guérin chez le rat. *C. r. Soc. Biol.*, **153**, 919.

HELLSTRÖM, I., HELLSTRÖM, K. E., SJÖGREN, H. D. & WARNER, G. A. (1971) Demonstration of Cell-mediated Immunity to Human Neoplasms of Various Histological Types. *Int. J. Cancer*, **7**, 1.

HERBERMAN, R. B. (1973) Immune Responses to Virus Induced Experimental Leukaemia and to Human Leukaemia. Proc. Miles Seventh International Symp. *The Role of Immunological Factors in Virus and Oncogeneic Process*. In the press.

IKONOPISOV, R. L., LEWIS, M. G., HUNTER-CRAIG, I. D., BODENHAM, D. C., PHILLIPS, T. M., COOLING, C. I., PROCTOR, J., HAMILTON FAIRLEY, G. & ALEXANDER, P. (1970) Auto-Immunization with Irradiated Tumour Cells in Human Malignant Melanoma. *Br. med. J.*, ii, 752.

LEUKEMIA STUDY GROUP A, U.S.A. (1973) B.C.G. Vaccination for the Maintenance of Remission in Childhood Leukemia. Personal communication.

MANTEL, N. (1966) Evaluation of Survival Data and Two New Rank Order Statistics Arising in its Consideration. *Cancer Chemother. Rep.*, **50**, 3, 163.

MATHÉ, G. (1969) Approaches to the Immunological Treatment of Cancer in Man. *Br. med. J.*, iv, 7.

MATHÉ, G., POUILLART, P. & LAPEYRAQUE, F. (1969) Active Immunotherapy of L1210 Leukaemia Applied after the Graft of Tumour Cells. *Br. J. Cancer*, **23**, 814.

M.R.C. Report on the Treatment of Acute Lymphoblastic Leukaemia (1971). *Br. med. J.*, iv, 189.

OLD, L. J., BENACERAFF, B., CLARK, D. A., CARSWELL, E. A. & STOCKERT, E. (1961) The Role of the Reticuloendothelial System in the Host Reaction to Neoplasia. *Cancer Res.*, **21**, 1281.

PARR, I. (1972) Response of Syngeneic Murine Lymphomata to Immunotherapy in Relation to the Antigenicity of the Tumour. *Br. J. Cancer*, **26**, 174.

PETO, R. & PETO, J. (1972) Asymptotically Efficient Rank Invariant Test Procedures. *J. R. statist. Soc.*, Series A. **2**. In the press

POWLES, R. L. (1973) Immunotherapy for Acute Myelogenous Leukaemia. *Br. J. Cancer*, **28**, Suppl. I, 262.

POWLES, R. L., BALCHIN, L. A., HAMILTON FAIRLEY, G. & ALEXANDER, P. (1971) Recognition of Leukaemic Cells as Foreign Before and After Autoimmunization. *Br. med. J.*, i, 486.

POWLES, R. L. & GRANT, C. (1973) Some Properties of Cryopreserved Acute Leukaemia Cells. Cryobiology. In the press.

POWLES, R. L., KAY, H. E. M., MCELWAIN, T. J., ALEXANDER, P., CROWTHER, D., HAMILTON FAIRLEY, G. & PIKE, M. (1973) *Recent Results in Cancer Research; Investigation and Stimulation of Immunity in Cancer Patients*. Berlin-Heidelberg-New York: Springer-Verlag. In the press.

VIZA, D. C., BERNARD-DEGANI, O., BERNARD, C. & HARRIS, R. (1969) Leukaemic Antigens. *Lancet*, ii, 493.

WHITECAR, J. P., BODEY, G. P., FREIREICH, E. J., MCCREDIE, K. B. & HART, J. S. (1972) Cyclophosphamide (NSC-26271), Vincristine (NSC-67574), Cytosine Arabinoside (NSC-63878), and Prednisone (NSC-10023) (COAP) Combination Chemotherapy for Acute Leukaemia in Adults. *Cancer Chemother. Rep.*, **56**, 543.